# Design, Modeling, and Control of an Aurelia-Inspired Robot Based on SMA Artificial Muscles

**DOI:** 10.3390/biomimetics8020261

**Published:** 2023-06-15

**Authors:** Yihan Yang, Chenzhong Chu, Hu Jin, Qiqiang Hu, Min Xu, Erbao Dong

**Affiliations:** 1 CAS Key Laboratory of Mechanical Behavior and Design of Materials, Department of Precision Machinery and Precision Instrumentation, University of Science and Technology of China, Hefei 230026, China; yyhx@mail.ustc.edu.cn (Y.Y.);; 2 Department of Biomedical Engineering, City University of Hong Kong, Kowloon, Hong Kong

**Keywords:** bio-inspired robot, untethered underwater robot, jet propulsion robot, shape memory alloy (SMA) artificial muscle, central pattern generator (CPG)

## Abstract

This paper presented a flexible and easily fabricated untethered underwater robot inspired by Aurelia, which is named “Au-robot”. The Au-robot is actuated by six radial fins made of shape memory alloy (SMA) artificial muscle modules, which can realize pulse jet propulsion motion. The thrust model of the Au-robot’s underwater motion is developed and analyzed. To achieve a multimodal and smooth swimming transition for the Au-robot, a control method integrating a central pattern generator (CPG) and an adaptive regulation (AR) heating strategy is provided. The experimental results demonstrate that the Au-robot, with good bionic properties in structure and movement mode, can achieve a smooth transition from low-frequency swimming to high-frequency swimming with an average maximum instantaneous velocity of 12.61 cm/s. It shows that a robot designed and fabricated with artificial muscle can imitate biological structures and movement traits more realistically and has better motor performance.

## 1. Introduction

After millions of years of evolution, organisms usually have good motility, flexibility, and environmental adaptability, which have inspired the design of robots. In recent decades, researchers have developed many soft robots inspired by natural organisms or their organs, such as earthworms [1], caterpillars [2], octopuses [3], fishes [4], and starfishes [5]. A “dancer” in the ocean, jellyfish have a soft body, low noise and energy consumption, high swimming efficiency, and simple anatomical structures. Because of these unique characteristics, jellyfish are of interest to researchers in the field of bionic robotics research [6,7,8,9,10,11,12,13,14,15,16], showing great potential for lake detection, water quality monitoring, aquaculture, and marine defense.

Along with the development of materials science and robotics, an increasing number of researchers are paying attention to soft actuators designed and manufactured by smart materials, such as shape memory alloy (SMA), electroactive polymer (EAP), and ionic polymer metal composites (IPMC). These soft actuators are characterized by high flexibility, compatibility, and adaptability, compared with traditional actuators that typically use motors to precisely achieve motion and load requirements. Joseph [7] designed a biomimetic jellyfish robot utilizing IPMC actuators as the propulsive mechanism, but the robot can only swim at a maximum speed of 1.5 mm/s even if eight actuators were used. The force provided by IPMC is too small. Ko [8] used an electromagnetic actuation (EMA) system to design a jellyfish-like mini-robot. The robot had a body of 17 mm and a thickness of 0.5 mm and was actuated by four flexible fins. Marut [9] used electric motors to design a jet propulsion robot named “JetPRo”, which mimics the propulsion mechanism of the jellyfish and allows for 3D motion and high speed. JetPRo was actuated by an iris mechanism and had a body height of 7.9 cm and a diameter of 5.7 cm. The robot swims upwards with a maximum steady-state velocity of 11.6 cm/s. Wang [11] fabricated a transparent soft jellyfish robot using dielectric elastomer actuators (DEAs). This soft jellyfish robot can achieve vertical and horizontal movements in water by mimicking the actual pulsating rhythm of the Aurelia aurita. However, its motion range is limited. Table 1 shows the parameters of some underwater swimming jellyfish robots.

These jellyfish robots have focused on utilizing various artificial muscles to achieve jetting motions but ignored the motion control of the jellyfish robot, which made the motion mode of the jellyfish robot single. Our research interest is to fabricate an untethered jellyfish robot using SMA artificial muscle modules that are highly flexible. Therefore, we designed an untethered robot (Au-robot) that mimics the rowing propulsion movements of Aurelia in this paper. Au-robot can achieve a smooth transition from low-frequency swimming to high-frequency swimming and flexible spatial swimming motions with high swimming velocity and high biomimetic performance. We use a new control method that combines a central pattern generator (CPG) with an adaptive regulation (AR) heating strategy to achieve multimodal swimming and smooth swimming transitions for the Au-robot.

CPG is a neural circuit that can generate rhythmic patterns of neural activity without receiving input [17,18]. Biological studies have shown that central pattern generators (CPGs) are widespread in organisms for generating rhythmic signals for breathing, running, and jumping motions. CPGs have been widely used by researchers for robot control since their proposal [19,20,21,22,23]. There are two main mathematical models proposed by researchers [18]: the neuron CPG model and the nonlinear oscillator CPG model. The neuron CPG model simulates the action potential of biological neurons, including the H-H model, M-L model, Stein model, and Matsuoka model. The model based on nonlinear oscillators has three typical categories: the Kuramoto model, the Hopfield model, and the Van Boer model. Ijspeert constructed an adaptive nonlinear coupled control mechanism using the Hopfield model, which generates multiple coordinated rhythmic signals based on several control inputs with a simple structure, coupling parameter tuning, and stable sinusoidal output [17,24]. Ijspeert successfully used this model to enable a salamander-like robot to achieve rhythmic motions on land and in water [25].

The CPG signal is periodic, and its duty cycle is generally constant, but the heating and cooling processes of SMA artificial muscles are different. There, if the CPG signal is not modulated, it may lead to overheating of the artificial muscle. To obtain better control, a new control strategy called the adaptive regulation heating strategy is proposed based on the variation of SMA wire resistance based on our previous work [26]. The AR heating strategy can delay overheating of the SMA artificial muscle, realize the control of the bending angle, and increase the action frequency of the artificial muscle. In our combined control strategy, the SMA artificial muscle’s action frequency and the starting point of the heating process depend on the outputs of the CPG system, and the end point of the heating process depends on the AR heating strategy, which means that the control method can change the duty cycle of the CPG signal in real-time and effectively prevent the generation of thermal dead silence phenomenon of the SMA artificial muscle due to overheating.

## 2. Design and Fabrication

### 2.1. Design of the Au-Robot

**Figure 1:**
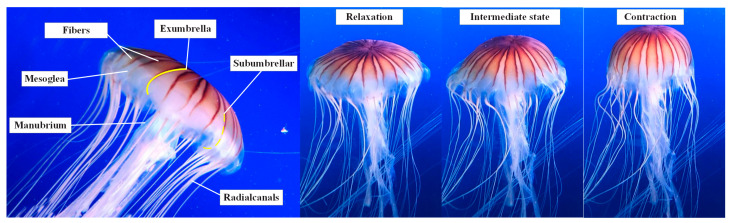
The details of Aurelia (photograph taken at Hefei Aquarium).

Jellyfish have a simple, soft, and symmetrical structure. The symmetrical property allows jellyfish to detect and prey in any direction. Most jellyfish are semitransparent or glassy, bell-shaped, and range from one inch to seven feet in diameter [27]. According to the fineness ratio, jellyfish can be separated into two categories called “Prolate” and “Oblate”. “Prolate” are usually smaller and swim faster, and “Oblate” are more energy efficient and attain wider bell diameters. Aurelia is one of the most common jellyfish, with a maximum diameter of up to 45 cm. Jellyfish move vertically mainly by pulse propulsion, while their horizontal movement largely depends on ocean currents and waves. Aurelia possesses radial muscles that potentially contribute to non-symmetric thrust, thereby causing turning maneuvers. Wider bell diameters make Aurelia swim more stable underwater. Some structural details about Aurelia are shown in Figure 1.

As shown in Figure 1, the top and bottom layers of the bell consist of a membrane called the epidermis, which is separated by mesoglea. The bell’s exterior part is referred to as the exumbrella, and the inner cavity is called the subumbrella. Aurelia’s action cycle is divided into two steps:

(i) Subumbrellar muscles shorten and contract the bell to expel water, which provides the thrust;
(ii) The bell regains its original configuration, using stored elastic energy.

Jellyfish do not have muscles to restore their initial bell structure but rely on the elastic energy stored in their structure during contraction. Fibers in the mesoglea are stretched during bell contraction as the walls thicken, allowing them to store elastic energy, which is used for bell relaxation [28].

**Figure 2:**
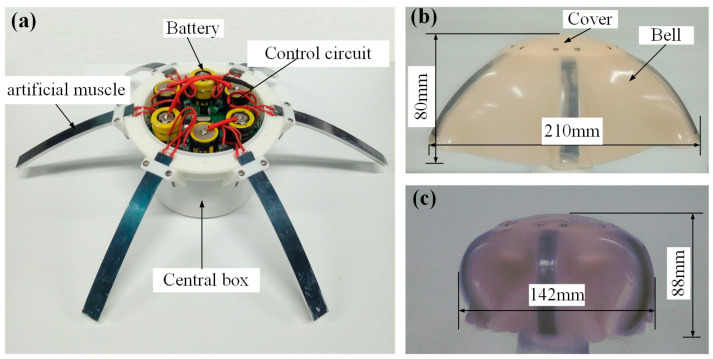
Structure of Au-robot. (**a**) Schematic of Au-robot; (**b**) Relaxation state of Au-robot; (**c**) Contraction state of Au-robot.

To mimic the structure of real Aurelia while implementing pulse and recovery processes, Au-robot was designed by modularly assembling the SMA artificial muscles, as shown in Figure 2.

A prototype of the Au-robot was constructed using six SMA artificial muscles symmetrically assembled on a central disc (Figure 2a). The central disc was fabricated by 3D printing using PLA plastic, which was used to accommodate the battery pack and the control circuit board. The battery pack consisted of six batteries (NITECORE NL18650A), which can provide a voltage of 20.8 V with a maximum current of 30 A. The Au-robot’s bell was made of elastic silicone with a thickness of 1 mm.

The weight of the prototype is 512 g, and the density is 1.031 g/cm^3^. Under the relaxation state (as shown in Figure 2b), the diameter is nearly 210 mm with a height of 80 mm. When the robot is in a contraction state (as shown in Figure 2c), the diameter is almost 142 mm with a height of 88 mm.

### 2.2. SMA Artificial Muscle

**Figure 3:**
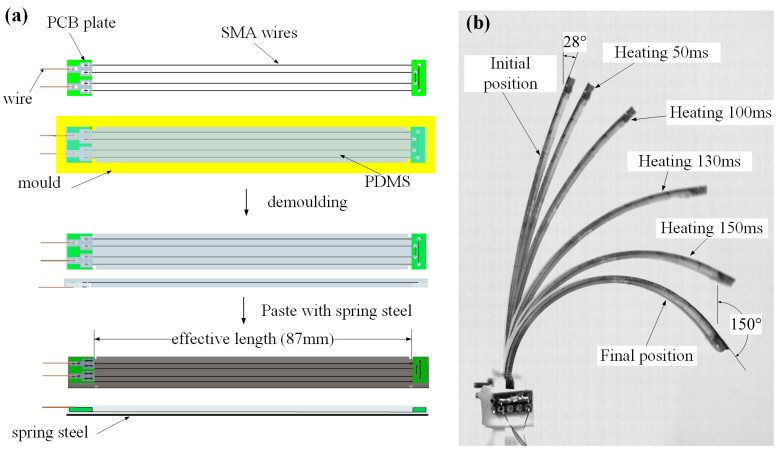
(**a**) Fabrication process of SMA artificial muscle. (**b**) The bending motion of the SMA artificial muscle heated by a voltage of 20 V.

SMA has a high power density ratio that enables a compact system size. Many researchers have presented various actuators based on SMA wires and used them in various applications [29,30,31,32,33,34,35,36,37,38]. To mimic the contraction-pulse propulsion mode of jellyfish, a novel artificial muscle based on SMA with new processes and parameters to implement reciprocal flapping motion was designed, which achieved a large bending deformation, as shown in Figure 3a. Benefiting from the characteristics of SMA, the SMA artificial muscle has a simple driving method, which causes bending deformation by applying voltage.

The SMA artificial muscle consists of three layers: the driving layer, the restoring layer, and the filling layer. The driving layer consists of SMA wires (0.15 mm diameter, transition temperature of 90 °C, and maximum deformation of 4.5%) and two PCB boards, SMA wires fixed on both sides of the PCB board. The restoring layer is a spring steel plate (thickness 0.1 mm), which plays a role in restoring the structure to its original state. The filling layer is the solidified polydimethylsiloxane (PDMS), which is used to bond the driving layer and the restoring layer together and ensure that the artificial muscle is waterproof. The detailed structural and physical parameters of the SMA artificial muscle are shown in Table 2. By loading voltage to the SMA artificial muscle, SMA wires generate heat to undergo a phase change, which generates force and displacement output. The contraction process of SMA wires is thus transformed into the bending motion of the artificial muscle due to the joint restriction of PDMS and the spring steel plate. The recovery process is achieved by the elastic potential energy stored in the spring steel plate.

We used a molding technique to fabricate the artificial muscle, and the molds were produced by a 3D printer using polylactic acid (PLA). A pre-strain was required when the drive layer was placed in the mold. The pre-strain gives an initial angle of the artificial muscle after demolding. In our experiments, we applied 0.5% pre-strain in the SMA wires, which induced the artificial muscle with an initial bending angle of 28° (Figure 3b). Additionally, the average resistance of the artificial muscles is close to 6 Ohm.

The artificial muscle can generate a two-dimensional reciprocal bending motion when heating the SMA wires. In the cooling process of the SMA wires, the restoring force of the spring steel restores the artificial muscle to its initial position. As shown in Figure 3b, the maximum bending termination angle of the artificial muscle is nearly 150° at a heating voltage of 20 V.

**Figure 4:**
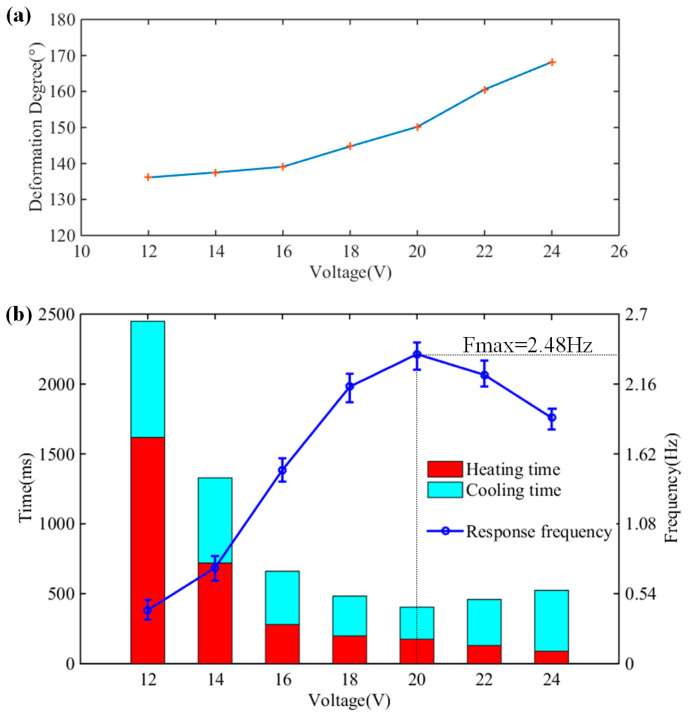
The performance of the SMA artificial muscle with different driving voltage. (**a**) Max bending angle; (**b**) response time and response frequency.

To obtain the optimal heating voltage, a series of experiments were carried out on the artificial muscle at different heating voltages. The bending angles of the artificial muscle were recorded by a high-speed camera (1000 frames/s). The relationship between the driving voltage and the maximum bending angle is shown in Figure 4a. The maximum bending angle of the artificial muscle increased with the increase of heating voltage. However, the overlarge heating voltage causes large shakes of the artificial muscle during the bending process, while too small a driving voltage leads to continuous heat accumulation in the artificial muscle and affects the frequency of the artificial muscle. Figure 4b shows the response frequency of the artificial muscle at different driving voltages. The details of the heating and cooling times of the artificial muscle are also presented. The heating time decreases with the increasing heating voltage. Because of the fluctuation and heat accumulation, the cooling time of the artificial muscle depends on whether the heating voltage is too large. The maximum response frequency of the artificial muscle is 2.48 Hz under a heating voltage of 20 V.

### 2.3. Material Parameter Tests

**Figure 5:**
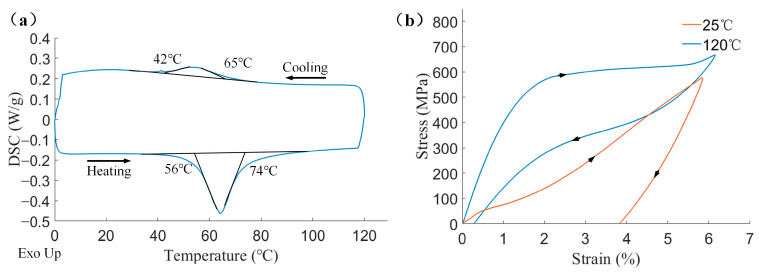
DSC and Uniaxial loading results of SMA. (**a**) DSC result; (**b**) Uniaxial loading result at 25 °C and 120 °C.

For the subsequent simulation analysis, we tested the basic characteristics of SMAs. A Differential Scanning Calorimeter (DSC) measurement is taken to determine the phase transition temperatures of the SMA specimens. The DSC result is shown in Figure 5a. Peaks in heating and cooling processes are recorded, from which we obtain the material’s zero-stress transformation temperatures easily. Heat-treated specimens are uniaxially loaded at two different temperatures (25 °C and 120 °C) to calculate the elastic stiffnesses at the parent phase and martensite. The results are shown in Figure 5b.

## 3. Thrust Model of the Au-Robot

The underwater motion of the Au-robot is affected by a variety of forces, such as gravity, buoyancy, thrust, and flow resistance. Among them, the magnitudes of gravity and buoyancy remain constant during the motion, while the points of action of the forces change at all times. Flow resistance is influenced by a variety of factors, such as robot shape, cross-section, and relative speed. Thrust is generated by the jet propulsion motion of the Au-robot. Since the SMA artificial muscles are soft and their structures change continuously during motion, it is difficult to build a complete motion model, so only the thrust model of the Au-robot underwater is modeled and analyzed. The method chosen to approximate the thrust production of the jellyfish robot combines a theoretical model with experimental measurements. The theoretical model was based on techniques developed for jetting jellyfish by Daniel [39,40,41]. Daniel believes that during the contraction phase, water is forcibly ejected or injected from the subumbrellar cavity through the velar aperture to provide thrust, and the thrust acts in a direction opposite to the motion of the ejected fluid. The thrust calculation method is given by the rate of momentum efflux:

(1) $$T=-u_{e}\frac{dm}{dt}$$

where T is the thrust, $u_{e}$ is the velocity of the ejected fluid, and *m* is the instantaneous mass of the medusa plus all water held in the subumbrellar cavity.

The velocity of the ejected fluid $u_{e}$ and the rate of mass change can be expressed by the rate of volume change of the Au-robot:

(2) $$\left\{\begin{array}{c} u_{e}=\frac{dV(t)}{dt}\cdot \frac{1}{A(t)}\\ dm=\rho dV(t) \end{array}\right.$$

where ρ is the density of water, *V*(*t*) is the instantaneous volume of Au-robot, and *A*(*t*) is the instantaneous sectional area of the velar aperture. Therefore, the thrust can be expressed as follows:

(3) $$T=-\frac{\rho }{A(t)}\cdot \frac{dV(t)}{dt}\left|\frac{dV(t)}{dt}\right|$$

To calculate *V*(*t*) and *A*(*t*), we have the following assumptions about the artificial muscle:

(1) The length of the neutral surface of the artificial muscle will not change during the bending process;
(2) During the bending of the artificial muscle, the contraction of SMA is the only direct factor of the system changes, and the deformation of the artificial muscle caused by the environmental temperature is negligible;
(3) The change in the distance between SMA and the neutral surface during the bending of the artificial muscle is negligible;
(4) The artificial muscle bending is approximately a standard arc.

**Figure 6:**
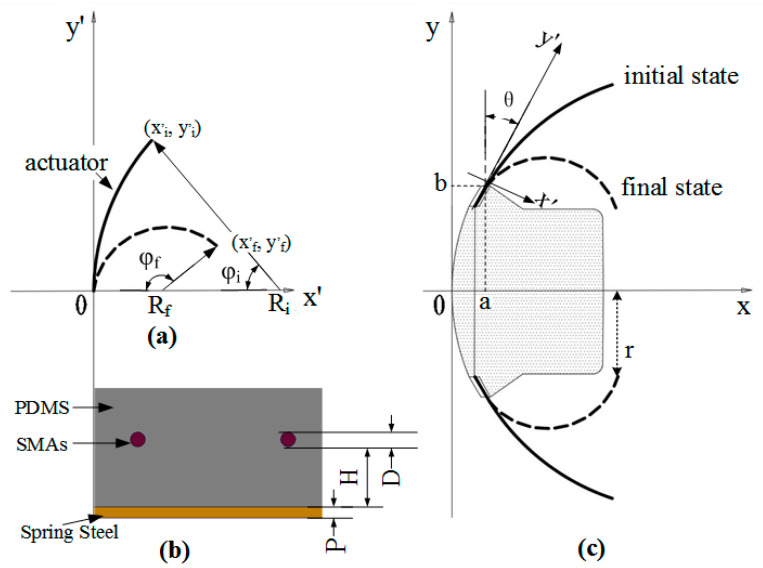
The geometric topologies of the artificial muscle and Au-robot. (**a**) Local coordinates; (**b**) the cross-section structure of the artificial muscle; (**c**) global coordinates.

Under the above assumptions, the location of the artificial muscle’s final position is shown in Figure 6c, and the coordinate of the final position and the analytical expression of the artificial muscle’s deformation can be obtained.

To facilitate the calculation of analytical expressions of artificial muscles, first set the starting point at the origin of the artificial muscle, next set the tangent as Y axis, as shown in Figure 6, so the center coordinate of the circular arc is (0, *R*(*t*)) in the X axis. In Figure 6b, D, H, and P are defined as the diameter of SMA, the distance between SMA and spring steel, and the thickness of spring steel are constant. *L*, *D*, *T*, and *H* belong to the drive structure parameters. According to the geometric relationship, it can be obtained:

(4) $$\left\{\begin{array}{c} L=R(t)\cdot \varphi (t)\\ L\cdot [1-\mu (t)]=[R(t)-(\frac{D}{2}+\frac{P}{2}+H)]\cdot \varphi (t)\\ {x_{i}}^{\prime }=R(t)\cdot [1-\cos\varphi (t)]\\ {y_{i}}^{\prime }=R(t)\cdot \sin\varphi (t) \end{array}\right.$$

where *L* is the effective length of the artificial muscle, *R* is the radius of the neutral layer of spring steel under the bending state, $\varphi \left(t\right)$ is the central angle, $\mu \left(t\right)$ is the contraction percentage of SMA, and $\left(x_{f}^{’},y_{f}^{’}\right)$ are the coordinates of the end of the artificial muscle. The coordinates of any point on the drive are $\left(x_{i}^{’},y_{i}^{’}\right)$, and there are:

(5) $$\left\{\begin{array}{c} {x_{i}}^{\prime }=\frac{D+P+2H}{2\mu (t)}[1-\cos\frac{2L\mu (t)}{D+P+2H}]\\ {y_{i}}^{\prime }=\frac{D+P+2H}{2\mu (t)}\sin\frac{2L\mu (t)}{D+P+2H} \end{array}\right.$$

(6) $${y_{i}}^{\prime }={\left(2{x_{i}}^{\prime }R(t)-{x_{i}}^{\prime 2}\right)}^{1/2}0\leq {x_{i}}^{\prime }\leq {x_{i}}^{\prime }$$

In this equation, *μ*(*t*) is the function of heating time and cannot be obtained directly. However, we assumed that the *μ*(*t*) is a simple function of the central angle $\varphi \left(t\right)$, and obtained by calculating Formula (4):

(7) $$\left\{\begin{array}{c} \mu (t)=k\varphi (t)\\ k=(D+P+2H)/2L \end{array}\right.$$

**Figure 7:**
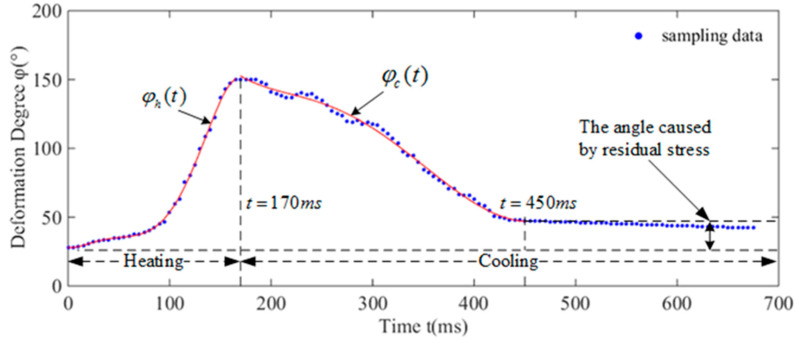
Relationship between the bending angle and time. In the first 170 ms, the SMAs were heated, increasing the deformation degree of the artificial muscle. In the next stage, the SMAs were cooled, corresponding to the decline of the curve.

Additionally, φ can be obtained by using a high-speed camera (1000 frames per second), and the result of the measurement is shown in Figure 7. We obtained a series of points about φ and *t*, and the relationship can be reached by five-order polynomial fitting with 95% confidence bounds. We gained two phases: heating ($\varphi_{h}\left(t\right)$) and cooling ($\varphi_{c}\left(t\right)$):

(8) $$\left\{\begin{array}{c} t=\left[\begin{array}{cccccc} t^{5} & t^{4} & t^{3} & t^{2} & t^{1} & 1 \end{array}\right]\\ \varphi_{h}(t)=P_{h}\cdot t\\ \varphi_{c}(t)=P_{c}\cdot t \end{array}\right.$$

where

$P_{h}\;$= [−1.2657197 × 10^−8^ 4.38787 × 10^−6^ −4.5698 × 10^−4^ 0.0177 −0.065655 27.8867],

$P_{c}\;$= [−1.594× 10^−10^ 3.09477 × 10^−7^ −2.2091 × 10^−4^ 0.07273 −11.4340 843.9239].

Through coordinate vector transformation in Figure 6a, the analytical formula of the circular arc in Figure 6c is obtained, that is, the analytical formula of the global coordinates of Au-robot:

(9) $$\left[\begin{array}{c} x_{i}\\ y_{i} \end{array}\right]=\left[\begin{array}{cc} \cos\theta & -\sin\theta \\ \sin\theta & \cos\theta \end{array}\right]\left[\begin{array}{c} {x_{i}}^{\prime }\\ {y_{i}}^{\prime } \end{array}\right]+\left[\begin{array}{c} a\\ b \end{array}\right]$$

where *θ* is the installation angle of the artificial muscle, and (*a*, *b*) is the fixed point coordinates of the artificial muscle.

The volume of Au-robot can be divided into two parts: 0≤x≤a and $a\leq x\leq x_{i}$. For the first part, it remains constant *V*_1_ during Au-robot movement, and in the second part, *V*_2_ can be regarded as a rotating body of $y=f\left(x\right)$ as the bus bar:

(10) $$V_{2}=\int_{a}^{x_{i}}\pi f^{2}(x)dx$$

(11) $$V=V_{1}+V_{2}$$

The cross-section radius of Au-robot is:

(12) $$\left\{\begin{array}{c} r=y_{i}={x_{i}}^{\prime }\sin\theta +{y_{i}}^{\prime }\cos\theta +b\\ A(t)=\pi r^{2} \end{array}\right.$$

The nomenclatures in all formulas in this section are given in Table 3.

**Figure 8:**
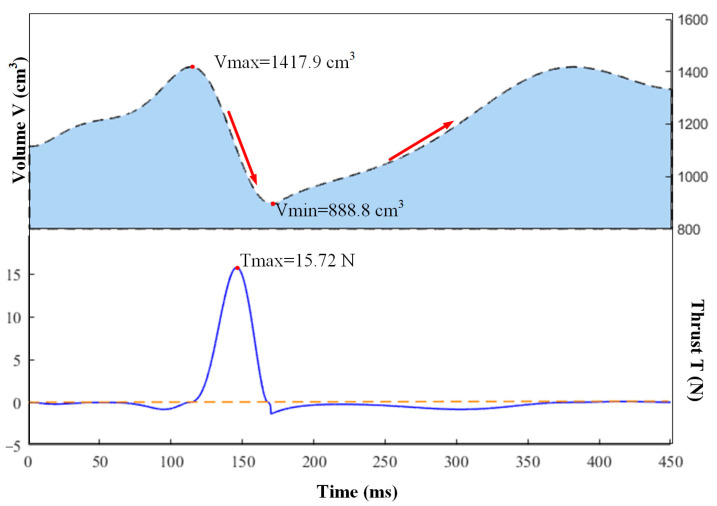
The thrust and volume of Au-robot vary with time. In an impulse period, the heating time is approximately 170 ms. The thrust peaked at the falling edge of the volume.

By using MATLAB simulation based on the above mathematical model, we were capable of obtaining the volume and thrust data of the Au-robot, which vary with time, as shown in Figure 8. Meanwhile, we revealed that the thrust of Au-robot is of impulse type and has a maximum value of 15.72 N. In one pulse period of about 450 ms, the volume of the Au-robot repeated changes two times and ranged between 1417 cm^3^ and 888.8 cm^3^. The thrust peaked at the falling edge of volume and became a negative value close to zero at the rising edge of volume, which proved that the thrust exerted an opposite effect in the process of Au-robot swimming.

## 4. Control Strategy

### 4.1. Central Pattern Generators Controller

In this part, we modified Ijspeert’s CPG model by redefining the output of the model. The mathematical description of the remodeling CPG model is as follows:

(13) $$\left\{\begin{array}{l} {\dot{\theta }}_{i}=2\pi f_{i}+\sum_{j}r_{j}w_{ij}\sin(\theta_{j}-\theta_{i}-\varphi_{ij})\\ \ddot{r}=a_{i}(\frac{a_{i}}{4}(R_{i}-r_{i})-{\dot{r}}_{i})\\ x_{i}=r_{i}\cos(\theta_{i})\\ S_{i}=\left\{\begin{array}{cc} 0 & x_{i}\leq 0\\ 1 & x_{i}>0 \end{array}\right. \end{array}\right.$$

While $\theta_{i}$ and $r_{i}$ are the phase and the amplitude state variables of oscillator *i*, respectively, $f_{i}$ and $R_{i}$ are defined as the anticipant frequency and amplitude, respectively. Couplings between oscillators are defined by the weights $\omega_{ij}$, and the phase biases $\varphi_{ij}$ is defined as the final difference of phase between oscillator *i* and *j*, $\varphi_{ij}=\lim\left(\theta_{i}-\theta_{j}\right)$ and $\varphi_{ij}=-\varphi_{ji}$. $a_{i}$ is a positive constant, and it determines the convergence time from $\theta_{i}$, $r_{i}$ to $f_{i}$, $R_{i}$. A positive oscillatory signal, $x_{i}$, represents the intermediate quantity of system outputs $S_{i}$.

It is worth noting that the greater the $\omega_{ij}$ means the faster the convergence of phase biases. However, overlarge $\omega_{ij}$ will result in a smooth system when the parameters of the system change. On the other hand, overlarge $a_{i}$ causes a similar problem with $\omega_{ij}$. In this paper, the coupling weight $\omega_{ij}$ among neighboring oscillators, and the positive constant $a_{i}$, are defined as constant values, $\omega_{ij}$ set as 200 and $a_{i}$ set as 30. Because $S_{i}$ is defined as a switch signal, we have binarization processing $S_{i}$ to 0 or 1. The CPG system is capable of generating different outputs when the input variables are changed.

**Figure 9:**
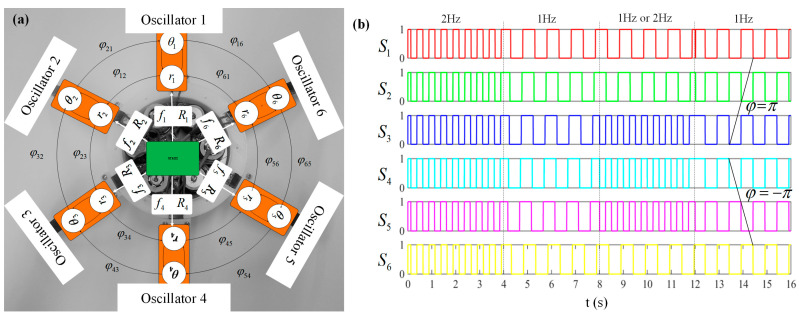
Topology of the CPG system’s oscillators network. (**a**) Circular CPG system net; (**b**) outputs of all oscillator subsystems.

As shown in Figure 9, we built a circular CPG system net that includes six oscillator subsystems numbered Oscillators 1 to 6, which correspond to the six fins of the Au-robot. The topology of the network shows the coupling relationship between oscillators.

Figure 9b shows the outputs of all oscillator subsystems. All the initial states of the subsystems have been set to 0. To prove the effectiveness of the CPG system, we changed the system input parameters, as shown in Table 4.

As seen in Figure 9b, the frequency of output signals was changed at 4 s, which proved that the CPG system could work at different frequencies. Between 8 and 12 s, the outputs of the CPG system show that the oscillator subsystems can work at different frequencies at the same time. Between 12 and 16 s, the phase bias of each oscillator subsystem is effective. The results show that the system output can timely respond to the parameters change and are continuous when the frequency and phase biases are changed. It is worth noting that Au-robot with different frequencies is reflected in the velocity of Au-robot’s swimming. The frequency differences among Au-robot fins realize the 3D swimming of the robot, and the phase biases of fins represent the waveform propulsion of the robot.

### 4.2. Signal Modulation by Adaptive Regulation Heating Strategy

**Figure 10:**
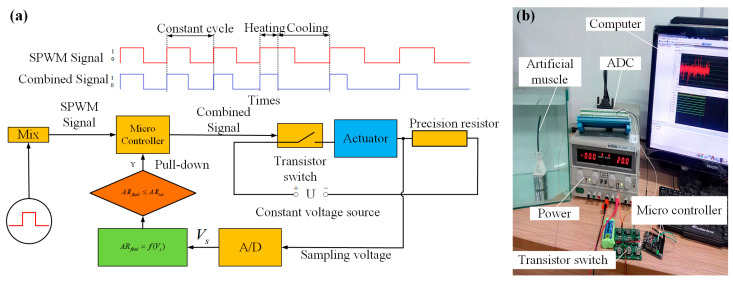
AR heating strategy. (**a**) Schematic of the signal modulation. (**b**) Experimental establishment of obtaining the regularity of variation in the resistance of the SMA wires.

The schematic of the AR heating strategy is shown in Figure 10a; a constant precise resistor was a series to the SMA artificial muscle. Along with the phase transformation of the SMA wires, a rapid fluctuation will occur in their resistance. An experimental test was designed, as shown in Figure 10b, to obtain the rule of fluctuation by sampling the voltage on the precision resistor. The desired sampling voltage is given by the following equation:

(14) $$V_{S}=R_{s}V_{i}/(R_{s}+R_{i}\times \varepsilon )$$

(15) $$R_{i}=(V_{i}-V_{s(t=0)})R_{s}/V_{s(t=0)}$$

In the above equation, $V_{s}$ is the theoretical sampling voltage, $R_{s}$ is the resistance of the precision resistor. $R_{i}$ is the initial resistance of the wires, and $V_{i}$ is the heating voltage. ε is the coefficient for the resistance ratio, r is the resistance of the wires in real-time, and ε is defined by the following equation:

(16) $$\varepsilon =r/R_{i}$$

**Figure 11:**
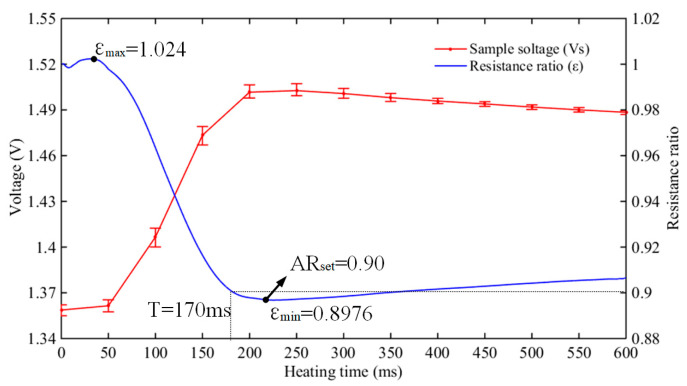
The resistance ratio of the SMA wires in the heating process.

By using a high-speed camera (1000 frames per second), the bending motion of the artificial muscle was recorded to analyze the relationship between the bending angle and the sampling voltage, as shown in Figure 11. We obtained the variation curve of the resistance ratio, and the maximum and minimum values are 1.024 and 0.8976. AR_set_ is a constant as a threshold, and the value of AR_set_ depends on the desired bending angle of the artificial muscle. In a sense, it determines the final state of the SMA artificial muscle. In our combined control strategy, AR_set_ is set to 0.90. When Au-robot moves, its built-in MCU will monitor the changes of ε at all times; the real-time ε is defined as AR_feed_, and the CPG signal would be pulled down while AR_feed_ < AR_set_, which means that the AR heating strategy changes the duty ratio of the CPG signal in real-time, avoids overheating, and makes the bending angle of the SMA artificial muscle controllable.

## 5. Experimental Results of the Au-Robot

The above proposed structural design and control strategy aimed to produce multiple-type motions of Au-robot. To prove their effectiveness, a series of tests were conducted (Supplemental Material Video S1).

**Figure 12:**
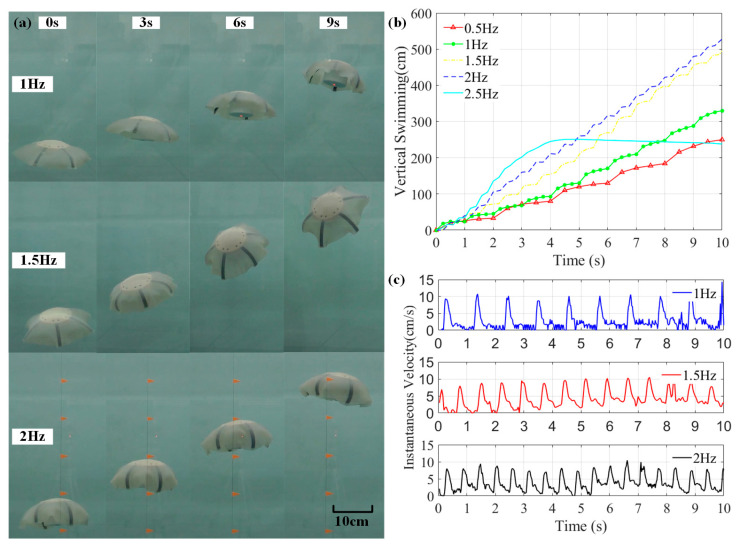
Vertical motions of Au-robot with 1 Hz, 1.5 Hz, and 2 Hz CPG signals. (**a**) Vertical motion, (**b**) the displacements, and (**c**) the instantaneous velocities.

In the first test, we were only concerned with the one-dimensional (vertical) velocity of the Au-robot. Figure 12a shows the vertical motion of the Au-robot under the CPG signals with different frequencies. All fins of Au-robot have the same frequency and no phase difference. Figure 12b,c show the displacements and instantaneous velocities of the vertical motion under different frequencies for the same time. From the experiment results, it is determined that the average maximum instantaneous velocities of Au-robot under different frequencies are similar, which are: 10.23 cm/s under 1 Hz CPG signal, 10.36 cm/s under 1.5 Hz, and 9.58 cm/s under 2 Hz. The result shows that different frequency has less influence on the maximum instantaneous velocity and more influence on the average velocity, while too high frequency will cause the overheating of the artificial muscle and reduce the average velocity of the Au-robot.

**Figure 13:**
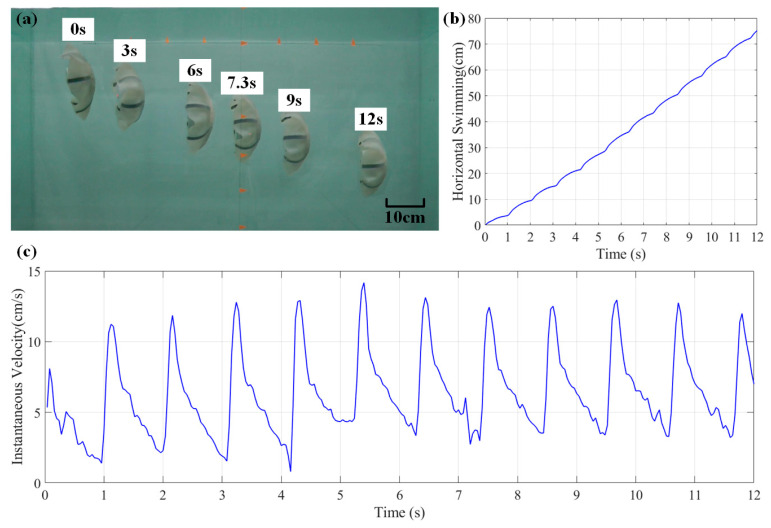
Horizontal motion of Au-robot with 1 Hz CPG signals. (**a**) Horizontal motion; (**b**) the displacement; (**c**) the instantaneous velocity.

Figure 13 shows the horizontal motion of the Au-robot. In horizontal swimming, the gravity of the robot causes it to shift 26% in the vertical direction and resulting in a faster velocity. When only horizontal displacement is considered, as shown in Figure 13b,c, the average maximum instantaneous velocity and average horizontal velocity are 12.61 cm/s and 7.94 cm/s, respectively.

**Figure 14:**
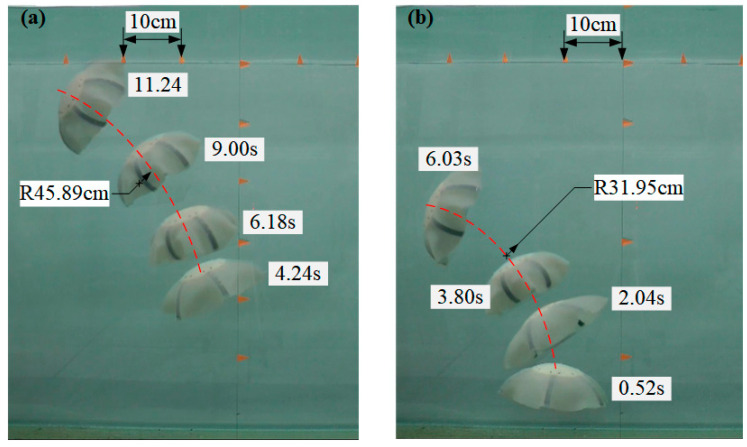
Turning motion. (**a**) Different action frequencies of fins; (**b**) some fins are stationary.

As shown in Figure 14, we tested two three-dimensional turning motions of the Au-robot. In Figure 14a, by changing the driving frequencies of the fins (three of the fins were set as 1 Hz, and the other fins were set as 1.5 Hz), the Au-robot produced a turning motion with a radius of 45.89 cm. In Figure 14b, three fins were set as static, and the other three were set as 1.5 Hz. In this case, the turning radius of the robot reached 31.95 cm.

## 6. Conclusions

This paper presents the design and fabrication of a new jellyfish robot inspired by Aurelia. To realize the pulse and recovery processes of Aurelia, a novel artificial muscle with new processes and parameters based on SMA is designed, which achieves a large bending deformation and meets the requirements of jet propulsion. Six radially symmetrically distributed SMA artificial muscles are used as fins of the jellyfish robot (Au-robot), each fin controlled by a new method that combines central pattern generators with an adaptive regulation heating strategy. The new control method can delay overheating of the SMA artificial muscle, control the bending angle and increase the action frequency of the SMA artificial muscle. This control method can generate CPG signals with different frequencies and phases with fewer inputs, which can effectively realize the robot swimming in different modes underwater. Compared with several jellyfish robots mentioned in the introduction, Au-robot has better comprehensive performance, such as quicker speed, realizing three-dimensional motion, and no external power supply. The robot can swim with a maximum instantaneous velocity of 12.61 cm/s, nearly 1.43 times the body length. Au-robot shows good motion characteristics and bionic performance.

Although the control method can generate different frequency and phase signals, it is still difficult to achieve more complex and accurate robot movements underwater due to the lack of sensing feedback from the robot. Future work can involve integrating more sensors into the robot, aimed at realizing autonomous cruise or human interaction. It is necessary to look for suitable applications, such as the exploration and development of lake sources, marine defense, water quality monitoring, and aquaculture. It is also feasible to replace jellyfish in the oceanarium and approach thousands of families. Numerous other challenges certainly remain, such as an optimized design and fabrication of the jellyfish robot, establishing a methodology to model the dynamic behavior of the robot during swimming, and maintaining the stability of swimming.

## Figures and Tables

**Table 1:** Comparisons of different jellyfish robots.

	Drive Method	Untethered	3D Swimming	The Size of Umbrella (cm)	Velocity (mm/s)
Bioinspired jellyfish [6]	Biosynthetic actuation	yes	no	0.9	2.4
Jellyfish-inspired vehicle [7]	IPMC	no	no	15	1.5
Jellyfish-like mini-robot [8]	EMA	yes	yes	1.7	-
JetPro [9]	motor	no	no	5.7	116
Soft Milliswimmer [10]	Magnetically-Actuated	yes	yes	0.4	50
Jellyfish Robot [11]	DEAs	no	yes	10.4	6.3
Jellyfish Robot [12]	SMA	no	yes	21	60
Jellyfish-inspired robot [13]	DE and IPMC	no	yes	12	4.8
Au-robot	SMA	yes	yes	21	126.1

**Table 2:** Parameters of SMA artificial muscle.

Items	Norms
Driving layer	4 × Φ0.15 mm × 87 mm
Restoring layer	100 mm × 10 mm × 0.15 mm (length × width × height)
Filling layer	100 mm × 10 mm × 2 mm (length × width × height)
The distance between driving layer and restoring layer	1.5 mm

**Table 3:** Nomenclatures and values.

Nomenclatures	Explanation or Values
T= thrust $\mu_{e}\;$= velocity of ejected fluid, V= volume of Au-robot A= sectional area of velar aperture r= radius cross-section φ= radius angle of the artificial muscle μ= contraction percentage R= radius of the artificial muscle	Time-varying
ρ= density of water	1000 kg/m^3^
L= effective length of the artificial muscle	80 mm
D= diameter of SMAs	0.15 mm
H= distance between SMAs and steel spring	1.5 mm
P= thickness of steel spring	0.1 mm
θ= installation angle of the artificial muscle	15°
k= ratio coefficient of proportionality μ,φ	(D+P+2H)/2L
$\left(x_{f}^{’},y_{f}^{’}\right)\;$= the end coordinate of the artificial muscle	In local coordinate system
$\left(x^{\prime },y^{\prime }\right)\;$= any point coordinates of the artificial muscle	In local coordinate system
$\left(x,y\right)\;$= any point coordinates of the artificial muscle	In global coordinate system
$P_{h}\;$= coefficient matrix of heating process	[−1.26572 × 10^−8^ 4.38787 × 10^−6^ −4.5698 × 10^−4^ 0.0177 −0.065655 27.8867]
$P_{c}\;$= coefficient matrix of cooling process	[−1.594 × 10^−10^ 3.09477 × 10^−7^ −2.2091 × 10^−4^ 0.07273 −11.4340 843.9239]

**Table 4:** Parameters of the CPG system.

Time/s	$\mathrm{Parameters}\;\left(\varphi_{ij}=-\varphi_{ji},/rad\right)$							
	*f*/Hz	*R*/V	$\varphi_{12}$	$\varphi_{23}$	$\varphi_{34}$	$\varphi_{45}$	$\varphi_{56}$	$\varphi_{61}$
0~4	2	1	0	0	0	0	0	0
4~8	1	1	0	0	0	0	0	0
8~12	1 or 2	1	0	0	0	0	0	0
12~16	1	1	π	π	0	−π	−π	0

## Data Availability

Not applicable.

## Supplementary Materials

The following supporting information can be downloaded at: https://www.mdpi.com/article/10.3390/biomimetics8020261/s1, Video S1: Underwater motions of the Au-robot.

## References

[1] Tang Z., Lu J., Wang Z., Chen W., Feng H. (2020). Design of a new air pressure perception multi-cavity pneumatic-driven earthworm-like soft robot. Auton. Robot..

[2] Lin H.T., Leisk G.G., Trimmer B. (2011). GoQBot: A caterpillar-inspired soft-bodied rolling robot. Bioinspiration Biomim..

[3] Wu Q., Yang X., Wu Y., Zhou Z., Wang J., Zhang B., Luo Y., Chepinskiy S.A., Zhilenkov A.A. (2021). A novel underwater bipedal walking soft robot bio-inspired by the coconut octopus. Bioinspir. Biomim..

[4] Clark A.J., Tan X.B., McKinley P.K. (2015). Evolutionary multiobjective design of a flexible caudal fin for robotic fish. Bioinspir. Biomim..

[5] Mao S., Dong E., Jin H., Xu M., Zhang S., Yang J., Low K.H. (2014). Gait Study and Pattern Generation of a Starfish-Like Soft Robot with Flexible Rays Actuated by SMAs. J. Bionic Eng..

[6] Nawroth J.C., Lee H., Feinberg A.W., Ripplinger C.M., McCain M.L., Grosberg A., Dabiri J.O., Parker K.K. (2012). A tissue-engineered jellyfish with biomimetic propulsion. Nat. Biotechnol..

[7] Najem J., Sarles S.A., Akle B., Leo D.J. (2012). Biomimetic jellyfish-inspired underwater vehicle actuated by ionic polymer metal composite actuators. Smart Mater. Struct..

[8] Ko Y., Na S., Lee Y., Cha K., Ko S.Y., Park J., Park S. (2012). A jellyfish-like swimming mini-robot actuated by an electromagnetic actuation system. Smart Mater. Struct..

[9] Marut K., Stewart C., Michael T., Villanueva A., Priya S. (2013). A jellyfish-inspired jet propulsion robot actuated by an iris mechanism. Smart Mater. Struct..

[10] Ren Z., Wang T., Hu W., Sitti M. (2019). A Magnetically-Actuated Untethered Jellyfish-Inspired Soft Milliswimmer. Robot. Sci. Syst. Xv.

[11] Wang Y., Zhang P., Huang H., Zhu J. (2022). Bio-Inspired Transparent Soft Jellyfish Robot. Soft Robot..

[12] Almubarak Y., Punnoose M., Maly N.X., Hamidi A., Tadesse Y. (2020). KryptoJelly: A jellyfish robot with confined, adjustable pre-stress, and easily replaceable shape memory alloy NiTi actuators. Smart Mater. Struct..

[13] Wang S.B., Chen Z. (2022). Modeling of Two-Dimensionally Maneuverable Jellyfish-Inspired Robot Enabled by Multiple Soft Actuators. IEEE-Asme Trans. Mechatron..

[14] Yeom S.W., Oh I.K. (2009). A biomimetic jellyfish robot based on ionic polymer metal composite actuators. Smart Mater. Struct..

[15] Wang T., Joo H.J., Song S., Hu W., Keplinger C., Sitti M. (2023). A versatile jellyfish-like robotic platform for effective underwater propulsion and manipulation. Sci. Adv..

[16] Xu N.W., Townsend J.P., Costello J.H., Colin S.P., Gemmell B.J., Dabiri J.O. (2020). Field Testing of Biohybrid Robotic Jellyfish to Demonstrate Enhanced Swimming Speeds. Biomimetics.

[17] Ijspeert A.J. (2008). Central pattern generators for locomotion control in animals and robots: A review. Neural Netw..

[18] Yu J., Tan M., Chen J., Zhang J. (2014). A Survey on CPG-Inspired Control Models and System Implementation. IEEE Trans. Neural Netw. Learn. Syst..

[19] Liao X., Zhou C., Wang J., Fan J., Zhang Z. (2023). A Wire-driven Elastic Robotic Fish and its Design and CPG-Based Control. J. Intell. Robot. Syst..

[20] Wang M., Dong H., Li X., Zhang Y., Yu J. (2019). Control and Optimization of a Bionic Robotic Fish Through a Combination of CPG model and PSO. Neurocomputing.

[21] Sun G., Sartoretti G. (2022). Joint-Space CPG for Safe Foothold Planning and Body Pose Control During Locomotion and Climbing. IEEE Robot. Autom. Lett..

[22] Yao C., Liu C., Xia L., Liu M., Chen Q. (2022). Humanoid adaptive locomotion control through a bioinspired CPG-based controller. Robotica.

[23] Xu Z., Fang Q., Liu C., Chen Q. (2023). Central Pattern Generator with Defined Pulse Signals for Compliant-Resistant Control of Biped Robots. Biomimetics.

[24] Ijspeert A.J. (2001). A connectionist central pattern generator for the aquatic and terrestrial gaits of a simulated salamander. Biol. Cybern..

[25] Ijspeert A.J., Crespi A., Ryczko D., Cabelguen J.M. (2007). From swimming to walking with a salamander robot driven by a spinal cord model. Science.

[26] Jin H., Dong E., Alici G., Mao S., Min X., Liu C., Low K.H., Yang J. (2016). A starfish robot based on soft and smart modular structure (SMS) actuated by SMA wires. Bioinspir. Biomim..

[27] Xu N.W., Townsend J.P., Costello J.H., Colin S.P., Gemmell B.J., Dabiri J.O. (2021). Developing Biohybrid Robotic Jellyfish (Aurelia aurita) for Free-swimming Tests in the Laboratory and in the Field. Bio-Protoc..

[28] Megill W.M., Gosline J.M., Blake R.W. (2005). The modulus of elasticity of fibrillin-containing elastic fibres in the mesoglea of the hydromedusa Polyorchis penicillatus. J. Exp. Biol..

[29] Luo Q., Tong L., Bambach M., Rasmussen K.J., Khezri M. (2022). Active nonlinear buckling control of optimally designed laminated plates using SMA and PZT actuators. Thin-Walled Struct..

[30] Chen T., Zhang Y., Qiu S., Jiang J., Zhang Q., Zhang X. (2022). Experiments and Modeling of Machined Spring Rotary Actuators with Shape Memory Alloys. Materials.

[31] Hu Q.Q., Dong E.B., Sun D. (2021). Soft Gripper Design Based on the Integration of Flat Dry Adhesive, Soft Actuator, and Microspine. IEEE Trans. Robot..

[32] Jin H., Dong E., Xu M., Yang J. (2020). A Smart and Hybrid Composite Finger with Biomimetic Tapping Motion for Soft Prosthetic Hand. J. Bionic Eng..

[33] Jin H., Dong E., Xu M., Xia Q., Liu S., Li W., Yang J. (2018). Tunable smart digital structure (SDS) to modularly assemble soft actuators with layered adhesive bonding. Smart Mater. Struct..

[34] Wang Z., Hang G., Li J., Wang Y., Xiao K. (2008). A micro-robot fish with embedded SMA wire actuated flexible biomimetic fin. Sens. Actuators A-Phys..

[35] Wang W., Yu C.Y., Abrego Serrano P.A., Ahn S.H. (2020). Shape Memory Alloy-Based Soft Finger with Changeable Bending Length Using Targeted Variable Stiffness. Soft Robot..

[36] Wang W., Ahn S.H. (2017). Shape Memory Alloy-Based Soft Gripper with Variable Stiffness for Compliant and Effective Grasping. Soft Robot..

[37] Sun Y.L., Lueth T.C. (2023). Enhancing Torsional Stiffness of Continuum Robots Using 3-D Topology Optimized Flexure Joints. IEEE-Asme Trans. Mechatron..

[38] Sun Y.L., Liu Y.Q., Lueth T.C. (2022). Optimization of Stress Distribution in Tendon-Driven Continuum Robots Using Fish-Tail-Inspired Method. IEEE Robot. Autom. Lett..

[39] Daniel T.L. (1983). Mechanics and Energetics of Medusan Jet Propulsion. Can. J. Zool. Rev. Can. De Zool..

[40] Battista N., Gaddam M.G., Hamlet C.L., Hoover A.P., Miller L.A., Santhanakrishnan A. (2022). The Presence of a Substrate Strengthens the Jet Generated by Upside-Down Jellyfish. Front. Mar. Sci..

[41] Neil T.R., Askew G.N. (2018). Jet-paddling jellies: Swimming performance in the Rhizostomeae jellyfish Catostylus mosaicus. J. Exp. Biol..

